# The Cognitive Thalamus as a Gateway to Mental Representations

**DOI:** 10.1523/JNEUROSCI.0479-18.2018

**Published:** 2019-01-02

**Authors:** Mathieu Wolff, Seralynne D. Vann

**Affiliations:** ^1^Centre National de la Recherche Scientifique, INCIA, Unité Mixte de Recherche 5287, Bordeaux, France,; ^2^University of Bordeaux, INCIA, Unité Mixte de Recherche 5287, Bordeaux, France, and; ^3^School of Psychology, Cardiff University, Cardiff, CF10 3AT, United Kingdom

## Abstract

Historically, the thalamus has been viewed as little more than a relay, simply transferring information to key players of the cast, the cortex and hippocampus, without providing any unique functional contribution. In recent years, evidence from multiple laboratories researching different thalamic nuclei has contradicted this idea of the thalamus as a passive structure. Dated models of thalamic functions are being pushed aside, revealing a greater and far more complex contribution of the thalamus for cognition. In this *Viewpoints* article, we show how recent data support novel views of thalamic functions that emphasize integrative roles in cognition, ranging from learning and memory to flexible adaption. We propose that these apparently separate cognitive functions may indeed be supported by a more general role in shaping mental representations. Several features of thalamocortical circuits are consistent with this suggested role, and we highlight how divergent and convergent thalamocortical and corticothalamic pathways may complement each other to support these functions. Furthermore, the role of the thalamus for subcortical integration is highlighted as a key mechanism for maintaining and updating representations. Finally, we discuss future areas of research and stress the importance of incorporating new experimental findings into existing knowledge to continue developing thalamic models. The presence of thalamic pathology in a number of neurological conditions reinforces the need to better understand the role of this region in cognition.

## Introduction

For over half a century, learning and memory have been intimately associated with the hippocampal formation, often leaving the functional contribution of other brain regions overlooked. However, the thalamus also has a long-standing link to memory. Indeed, damage within this region invariably occurs in Korsakoff syndrome, one of the key symptoms of which is a dense amnesia (Kopelman et al., 2009). The co-occurring diencephalic damage in this condition was noted as early as the end of the 19th century (Gudden, 1896), with a more explicit link between diencephalic damage and memory subsequently made by Gamper (1928). However, it was not until later in the 20th century that these brain regions began to gain further interest, by which time there was already a widespread focus on the medial temporal lobe for memory function, following the reports of Patient H.M. (Delay and Brion, 1969; Victor et al., 1971; Scoville and Milner, 2000). Thus, although the severity of memory impairments is often largely comparable between temporal lobe and diencephalic amnesia (e.g., Hunkin et al., 1994; Shaw and Aggleton, 1995; Caulo et al., 2005), the role of the thalamus, and the diencephalon in general, has largely been disregarded. In a similar vein, the neural bases of reasoning, thought, and cognition are generally considered to be supported by the cortex, the prefrontal cortex (PFC) in particular (Donoso et al., 2014), with little, if any, role for subcortical areas. Thus, in terms of cognition, the thalamus has typically been viewed as a supporting member of the cast that acts simply as a relay for the main players (i.e., the hippocampus and the neocortex). Within these models, the thalamus has taken on a passive role, simply transferring information without providing any unique contribution to the system. In recent years, however, evidence has emerged that contradicts this idea of a passive relay and highlights a central role for the thalamus in cognition.

There are inherent difficulties in attempting to generate global models of thalamic functions because the thalamus is not a unitary structure. It comprises a large number of nuclei, each with different anatomical connectivity and functional properties. The idea of the thalamus as a heterogeneous structure with only a small number of nuclei supporting the canonical sensory-motor relay function was first established by the pioneering work of Guillery and Sherman (Sherman and Guillery, 1996). These authors further developed their model over the years, proposing a dichotomy of thalamic functions based primarily on the main type of afferents received by thalamic nuclei (for a comprehensive perspective of this work and of the major contribution of Ray Guillery, who sadly passed away last year, see Murray Sherman, 2018). Those nuclei receiving driver input (i.e., capable of directly eliciting neuronal activity) from the cortex are called higher-order nuclei and are thought to actively participate in cortical functioning (Sherman, 2016). In contrast, thalamic nuclei receiving driver input from subcortical regions are considered first-order thalamic nuclei (i.e., textbook relay thalamic nuclei). Other researchers characterized some thalamic nuclei as limbic on the basis of their connectivity with the cingulate cortex and their contribution to cognition rather than purely sensory-motor processes (Vogt and Gabriel, 1993). Both higher-order and limbic thalamic nuclei appear necessary for cognition (Vogt and Gabriel, 1993; Varela, 2014), but neither classification includes all nuclei that support this role; therefore, from a behavioral perspective, the term “cognitive thalamus” more accurately captures the essence of those thalamic nuclei that primarily support cognitive functions.

In this *Viewpoints* article, we will describe a revised model of the thalamus wherein, instead of merely acting as relays, thalamic nuclei contribute to cortical functioning and higher-order cognition, ranging from learning and memory to flexible adaptation. We will discuss the possibility that these apparently separate cognitive functions may indeed be supported by a more general role of the thalamus in maintaining and updating mental representations. The anterior thalamic nuclei (ATn) and the mediodorsal thalamus (MD) will serve as the main examples to illustrate this view. Given that extensive reviews of these thalamic nuclei are available (Bradfield et al., 2013a; Jankowski et al., 2013; Aggleton and Nelson, 2015; Dillingham et al., 2015a; Mitchell, 2015; Wolff et al., 2015a; Ouhaz et al., 2018; Pergola et al., 2018), our aim is not to give a detailed analysis of these areas but to highlight general functional principles that may transcend specific nuclei and so be relevant for the cognitive thalamus as a whole. We will also consider the role of corticothalamic versus thalamocortical projections and the integration of thalamocortical loops with other cortical and subcortical networks. In doing so, we hope to provide a general overview of the current state of knowledge and to identify areas where future research is needed.

### The cognitive thalamus

#### Learning and memory

Memory was probably the first cognitive function formally associated with the thalamus. Both the ATn and MD have been implicated in the memory impairments associated with the Korsakoff syndrome (Victor et al., 1971; Harding et al., 2000), but their individual contributions to learning and memory appear quite different (Bradfield et al., 2013a; Mitchell, 2015; Wolff et al., 2015a). Indeed, it had been proposed that a double dissociation existed between ATn and MD functions, with ATn supporting recollective memory and MD supporting familiarity-based memory (Aggleton and Brown, 1999). Although recent data suggest that this model does not entirely capture the mnemonic contribution of the MD (Danet et al., 2017), experimental manipulations in rodents have established clear distinctions between the types of memory processes supported by these different thalamic regions (Bradfield et al., 2013a; Wolff et al., 2015a).

ATn lesions in rodents produce striking impairments across spatial memory tasks, with the severity of deficit often comparable with that seen following hippocampal lesions (Warburton and Aggleton, 1999; Aggleton and Nelson, 2015). Impairments are found on reference and working memory, as well as path integration tasks (Warburton et al., 1997; Warburton and Aggleton, 1999; Frohardt et al., 2006). Thus, ATn lesions appear to disrupt the processing of environmental cues and the updating and monitoring of the animal's position within the environment. These spatial impairments are consistent with the electrophysiological properties of the ATn, as this structure contains a number of spatially responsive cells encoding information, such as orientation, spatial location, and running speed (Taube, 1995; Tsanov et al., 2011b; Jankowski et al., 2015; Laurens et al., 2016). In contrast, the recognition of single items does not appear to require the ATn, although the ATn may be important for reducing interference between multiple similar items (Law and Smith, 2012; Nelson and Vann, 2017). Furthermore, ATn lesion-induced impairments are found when animals are required to combine item memory with additional features, such as temporal order and location (Parker and Gaffan, 1997; Wilton et al., 2001; Wolff et al., 2006; Dumont and Aggleton, 2013; Nelson and Vann, 2014). While a similar pattern of deficits can be found following MD lesions (Cross et al., 2012) MD lesions also impair the ability to discriminate the temporal order of two items, whereas temporal order memory impairments following ATn lesions only emerge when multiple items are used (Mitchell and Dalrymple-Alford, 2005; Nelson and Vann, 2017).

Although MD lesions can disrupt performance on spatial memory tasks, this does not appear to arise from impairments of spatial memory per se, but rather from impairments of strategic aspects of the task (Hunt and Aggleton, 1998a). There is an ongoing assumption that MD is particularly important for working memory because of its connections with the PFC (Watanabe and Funahashi, 2012; Funahashi, 2013; Halassa and Kastner, 2017; Parnaudeau et al., 2018) and because delay-dependent cells are found in the primate MD (Funahashi, 2013). Cells displaying delay-dependent activity have also been found in the rodent MD, but the findings are far more variable with some studies showing activity at delays comparable with cells within the dorsomedial PFC (dmPFC) (Bolkan et al., 2017) and others showing no delay activity (Han et al., 2013; Miller et al., 2017). Although MD damage in primates disrupts working memory, these deficits are most often found in combination with other memory or executive deficits, suggesting that working memory itself may not be specifically compromised (Watanabe and Funahashi, 2012; Baxter, 2013). In rodents, data from delayed nonmatching-to-place tasks (i.e., spatial alternation) bring little support for the idea that the MD contributes to working memory. This task takes advantage of rodents' natural tendency to search in novel locations for food and requires rats to alternate between arms of a T-shaped maze, often for a reward. Although this behavioral task appears simple, it indeed relies on multiple cognitive processes and can be solved using several different strategies (Dudchenko, 2001). Deficits can thus reflect poor spatial memory, but several other factors can also affect performance, including impulsivity, motivation, reward-response associations, and interference sensitivity. When impaired performance is observed after MD damage, it has often been reported as transient or nonspecific (Stokes and Best, 1990; Hunt and Aggleton, 1991, 1998a; Alexinsky, 2001; Chauveau et al., 2005). Importantly, several experiments performed in different laboratories found delayed nonmatching-to-place performance to be unaffected after thalamic damage, even when damage was substantial and long delays were included (Neave et al., 1993; Hunt and Aggleton, 1998b; Mitchell and Dalrymple-Alford, 2005; Alcaraz et al., 2016b). Experimental data supporting the opposite view (i.e., a role for the MD in delayed nonmatching-to-place), mostly come from recent chemogenetic and optogenetic interventions conducted in mice in which impairments were found at longer delays (Parnaudeau et al., 2013; Bolkan et al., 2017), as well as during the acquisition of the nonmatching-to-place task (Parnaudeau et al., 2013). The apparent discrepancy between these findings and earlier studies may arise for a number of reasons. The most pronounced impairments in these mouse studies are found during longer delays in well-trained animals and thus might reflect impairments in additional factors, such as impulsivity. The specificity of viral spread within the thalamus may also be an issue in mice, and potential encroachment into adjacent thalamic nuclei, such as the ATn, could contribute to the findings (Hunt and Aggleton, 1998b; Mitchell and Chakraborty, 2013; Aggleton and Nelson, 2015; Wolff et al., 2015a), given that damage to the ATn, but not MD, severely impairs spatial working memory (Alcaraz et al., 2016b). Together, the overall picture appears to be that MD is not necessary for working memory but may contribute to additional aspects of task performance, such as delay monitoring or habit formation when animals are overtrained.

#### Shaping mental representations

Decades ago, Tolman (1948) coined the term “cognitive map” to refer to a highly organized knowledge database that allows flexible actions. Cognitive maps can be considered mental representations requiring the combination of external cues with internal states to generate accurate depictions of general rules and/or associative laws. These representations are vital for animals to successfully interact with the world (Ramos, 2014). Although it is possible to dissociate thalamic nuclei on the basis of their distinct cognitive functions (Mitchell and Dalrymple-Alford, 2005, 2006; Wolff et al., 2008b, 2015a, b; Bradfield et al., 2013a; Moreau et al., 2013), this does not contradict the idea of an overall involvement of the cognitive thalamus in shaping mental representations. For instance, while the ATn and the MD belong to distinct functional circuits, they are both considered important for directing attention to task-relevant behavioral features (Wolff et al., 2015b; Wright et al., 2015), which is required to build task-relevant mental constructs. Moreover, thalamic damage often impairs memory acquisition, suggesting further that forming meaningful representation requires thalamic integrity (Cermak et al., 1980; Vann and Aggleton, 2003; Wolff et al., 2008b; Marchand et al., 2014; Sweeney-Reed et al., 2014).

Even after initial learning is established, the thalamus continues to play an important role, possibly by monitoring and updating current information within a changing environment. For example, ATn damage appears to be particularly detrimental when elements of flexibility are required to solve ongoing challenges: the ability to reach a previously learned position from a new start is disproportionally impaired by ATn lesions (Wolff et al., 2008a), as is spatial alternation when the animal is released from opposite arms for sample and test trials (Warburton et al., 1997; Loukavenko et al., 2007, 2016). A common feature of these experimental situations is that animals must track changes in task demands and update their current frame of reference accordingly to maintain successful performance. The MD also appears to be particularly important when successful performance requires the update of action-outcome or stimulus-outcome associations, as shown in rodents (Corbit et al., 2003; Ostlund and Balleine, 2008; Bradfield et al., 2013a; Parnaudeau et al., 2015; Alcaraz et al., 2016b, 2018) and also in primates (Mitchell et al., 2007; Izquierdo and Murray, 2010; Browning et al., 2015; Chakraborty et al., 2016; Wicker et al., 2018).

Together, these data highlight a role for thalamic nuclei in monitoring, maintaining, and updating mental constructs, in contrast to previous views, which have emphasized the dominant role of cortical areas (Wilson et al., 2010, 2014; Markov et al., 2013). Increasing evidence indicates instead that close functional interactions between cortical and thalamic areas are essential to shape these representations to address ongoing challenges (Cross et al., 2012; Parnaudeau et al., 2013; Browning et al., 2015; Bolkan et al., 2017; Miller et al., 2017; Schmitt et al., 2017; Alcaraz et al., 2018; Marton et al., 2018). To better understand the nature of these interactions, it is necessary to consider specific features of the organization of thalamocortical circuits.

### The thalamocortical loop

**Figure 1.**
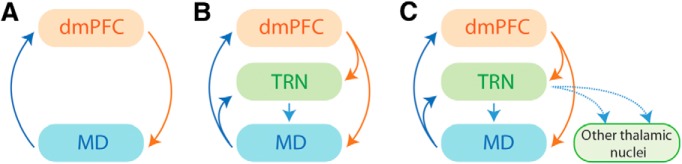
Different degrees of complexity for thalamocortical architecture. Basically defined as the reciprocal projections between prefrontal and thalamic areas (***A***), the thalamocortical loop includes an additional layer constituted by the reticular thalamic nucleus (TRN, ***B***). This area is one of the main sources of thalamic inhibition. Both thalamocortical and corticothalamic pathways send collaterals to the TRN. In addition, the TRN sends supplemental inhibitory projections to other thalamic nuclei (***C***), not included in the actual loop, thus opening this loop, which may allow gating of specific thalamocortical inputs (see also Fig. 3).

One hallmark of thalamocortical circuits is the reciprocity of projections between cortical and thalamic areas. This has been viewed as “reentry,” a process whereby two or more brain regions concurrently stimulate, and are stimulated by, each other. This reciprocal and parallel processing supports the synchronization of neuronal firing required for rapid neural integration. The binding of activity across a number of regions is thought to underpin the conscious processing of stimuli, which is necessary to form a unified mental construct (e.g., a scene or visual representation) (Tononi and Edelman, 1998; Tononi et al., 1998; Edelman and Gally, 2013). As a result of recent technical advances, it is now possible to selectively target projection-defined neurons, which has opened up new possibilities in assessing the functional role of thalamocortical *versus* corticothalamic pathways. Two recent studies have used this approach and have shown that reciprocal pathways between MD and the dmPFC can be functionally differentiated (Bolkan et al., 2017; Alcaraz et al., 2018). Thus, thalamocortical and corticothalamic pathways may play complementary but dissociable roles in cognition. Unlike thalamocortical projections, which are mostly ipsilateral, corticothalamic projections also provide substantial contralateral innervation at the thalamic level (Preuss and Goldman-Rakic, 1987; Négyessy et al., 1998; Bradfield et al., 2013a; Mathiasen et al., 2017). These projections include collaterals to the reticular thalamic nucleus, which in turn provides lateral inhibition for virtually any thalamic nucleus (Pinault, 2004; Halassa and Acsády, 2016) (Fig. 1). The functional relevance of this organization is discussed below.

#### Corticothalamic pathways: directing cognitive resources

A cardinal feature of higher-order thalamic nuclei is that they receive both a modulatory input and a driver input from the cortex (from layers 6 and 5, respectively) (Usrey and Sherman, 2018). This organization suggests important functional roles for corticofugal pathways, possibly implementing additional and indirect corticocortical routes through the thalamus (Jones, 1998; Sherman, 2005, 2012). This view is, however, largely speculative and mostly derived from neurophysiological studies of sensory-motor functions (Sherman, 2016). But even sensory mechanisms can contribute to cognition: they may be viewed as enabling abstraction of relevant information, thus helping to represent the external world in a meaningful way (Cudeiro and Sillito, 2006). Branching of thalamocortical pathways at the level of the reticular thalamic nucleus may enable the gating of salient thalamic inputs by minimizing the importance of those that are currently irrelevant (McCormick and von Krosigk, 1992; Zikopoulos and Barbas, 2007; Stillova et al., 2015), thus providing a possible mechanism of focused attention (Béhuret et al., 2015; Wimmer et al., 2015). This view is rooted in the ideas initially developed by Crick (1984) of the reticular thalamic nucleus acting as an attentional searchlight. Interestingly, increased modulation of corticothalamic pathways has been found to parallel increased attentional demand in humans (Jagtap and Diwadkar, 2016). The dynamic nature of the excitatory-inhibitory balance at the thalamic level depends on current behavioral demand, with critical dependence on corticothalamic pathways and their collaterals to the reticular thalamic nucleus (Crandall et al., 2015; Li and Ebner, 2016). It is thus possible that cortical projections to the thalamus directly adjust the gain and the tuning precision of thalamic cells as required by ongoing behavioral demands (Mease et al., 2014; Guo et al., 2017a).

Beyond their role in directing attentional resources, corticocothalamic pathways have also been linked to processes underpinning learning. For example, direct evidence for a causal involvement of corticothalamic pathways in learning has been reported in an appetitive Pavlovian conditioning task. Optogenetic manipulation of the projections from PFC to the paraventricular thalamic nucleus during task acquisition affected the conditioned response, highlighting a role for this pathway in the encoding of predictive environmental cues (Otis et al., 2017). Furthermore, dmPFC-to-MD pathways have been demonstrated to support upcoming choice either in a spatial working memory task (Bolkan et al., 2017) or when the retrieval of current goal value is required for successful responding (Alcaraz et al., 2018). Importantly, these corticothalamic pathways also promote behavioral flexibility (Nakayama et al., 2018), especially when rule switching is required (Marton et al., 2018). Collectively, these data suggest a central role for corticothalamic pathways in cognition, and their functional relevance seems to range from directing attention to solving cognitive challenges.

#### Thalamocortical pathways: more than a relay

Central to almost all definitions of thalamic function is the concept that this region is a “relay.” Even those nuclei that are considered to have a more cognitive role are still considered to be principally involved in relaying information either between cortical sites or between medial temporal lobe and neocortex. This description of thalamic function attributes little or no additional role for these nuclei other than acting as a waystation. However, this clearly underestimates and oversimplifies the role of the thalamus. The idea of thalamic regions monitoring and updating information and providing an active contribution rather than a passive relay is not in fact new. The MD was previously suggested to be involved in mediating cognitive aspects of odor-guided tasks rather than transmitting sensory information (Eichenbaum et al., 1980). The latter possibility was considered because the MD links the piriform cortex (the primary olfactory cortex) with the orbitofrontal associative cortex (Courtiol and Wilson, 2015). But even when using an odor-guided behavioral assay, it appears that task-related features, rather than purely sensory information, are represented by MD cells (Courtiol and Wilson, 2016).

More recently, other evidence has emerged that also supports the idea of nonrelay contributions of the thalamus. For example, Schmitt et al. (2017) recently showed that the MD is able to sustain cortical representations rather than relaying information. These data emphasize a role for the thalamus in controlling cortical connectivity to maintain rule representation (Halassa and Kastner, 2017; Nakajima and Halassa, 2017). A causal relationship between MD-PFC activity and social dominance behavior was also recently established, underscoring further the importance of thalamic inputs for cortical functions (Zhou et al., 2017). It should be noted that the importance of sustained thalamocortical activity during delay has been observed to instruct future actions in other thalamocortical circuits (Guo et al., 2017b), suggesting that sustaining cortical activity may constitute an essential role of thalamic inputs.

Like the MD, the ATn does not passively relay information. Instead these nuclei show long-term, input-dependent modification of their responses, which can amplify the convergent inputs from different sources (Tsanov et al., 2011a). Behavioral data are also consistent with a nonrelay function for the ATn: the behavioral effects of ATn lesions can be more pronounced than lesions disrupting any of their individual inputs (e.g., Aggleton et al., 1995; Warburton and Aggleton, 1999; Sziklas and Petrides, 2000; Wright et al., 2015; Powell et al., 2017), suggesting that no single pathway supports all cognitive aspects of ATn function. Therefore, it is unlikely that the ATn is a simple relay in the traditional sense, but instead integrates information from midbrain, diencephalic, hippocampal, and cortical regions (Tsanov et al., 2011a; Vann and Nelson, 2015; Mathiasen et al., 2017). This view is also supported by numerous studies showing the importance of ATn inputs for driving activity in their cortical target, the retrosplenial cortex. In rodents, ATn lesions disrupt a number of markers of activity in the retrosplenial cortex (Dupire et al., 2013; Mendez-Lopez et al., 2013; Aggleton and Nelson, 2015). Furthermore, the retrosplenial cortex is hypoactive in patients with thalamic damage (Reed et al., 2003). Importantly, retrosplenial cortex activity changes are not simply a result of deafferentation (Garden et al., 2009; Vann and Albasser, 2009; Frizzati et al., 2016), but likely reflect the loss of functional coupling between the ATn and retrosplenial cortex. Indeed, the close functional correspondence between the ATn, hippocampus, and retrosplenial cortex highlights the possible importance of the thalamus in synchronizing activity across multiple regions (Corcoran et al., 2016; Eichenbaum, 2017; Halassa and Kastner, 2017). Indeed, this function may be paramount for its role in updating existing representations.

### Beyond the thalamocortical loop

At this point, it seems appropriate to expand more broadly our views of thalamic functions, looking beyond the thalamocortical loop. How exactly do these loops integrate with other cortical and subcortical circuits?

#### Cortical integration

**Figure 2.**
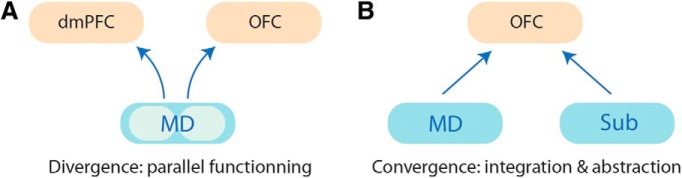
In addition to the reciprocity of projections, divergence (***A***) and convergence (***B***) are two prominent features of thalamocortical organization. Whereas the former underscores the possibility that multiple thalamic microcircuits act in parallel to achieve cognitive functions, the latter offers enhanced integrative properties. Regarding convergence, MD innervation of the dmPFC and the OFC originates from essentially separate neuronal populations (light blue), even though some MD cells branch to several prefrontal areas. Sub, Submedius thalamic nucleus.

Thalamocortical projections are both divergent and convergent (Rubio-Garrido et al., 2009). This feature of thalamocortical architecture provides an ideal basis for integration both within and across cortical regions (Fig. 2). Keeping with the example of the MD, several parallel thalamocortical pathways originate in MD and target distinct prefrontal areas (Groenewegen, 1988; Alcaraz et al., 2016a). In rodents, there is a clear topography as lateral MD neurons innervate the dorsal wall of the PFC while medial MD neurons predominantly contact its ventral wall. In contrast, MD cells located in the central segment essentially innervate the orbitofrontal cortex (OFC) (Alcaraz et al., 2016a; Murphy and Deutch, 2018). In addition to this distinct topography across MD, individual MD neurons also innervate several PFC regions, with collaterals contacting multiple cortical layers (Kuramoto et al., 2017b). Together, this illustrates the highly divergent nature of these thalamocortical projections.

By targeting different cell types and different neuronal compartments (Delevich et al., 2015; Collins et al., 2018), the MD can influence varying aspects of PFC functioning. For example, the MD projections to the dmPFC and the OFC have been proposed to support instrumental and Pavlovian associations, respectively (Ostlund and Balleine, 2008; Alcaraz et al., 2016a). An intriguing possibility is that such organization enables simultaneous instruction of distinct cortical areas through different pathways, further highlighting the multiple thalamic microcircuits that may act in parallel to support cognition (Rikhye et al., 2018).

Moving onto convergence, cortical regions are typically innervated by more than one thalamic nucleus. The PFC receives afferent projections from a large number of thalamic nuclei, including the intralaminar, midline, and anterior nuclei (Barbas et al., 1991). Taking the OFC as an example, the main thalamic efferents to the OFC originate from the MD and the much-less-known submedius thalamic nucleus (Yoshida et al., 1992; Alcaraz et al., 2016a; Kuramoto et al., 2017a). At present, the functional relevance of this type of organization is unclear, although both thalamic nuclei appear to support successful updating of stimulus-outcome associations, a cardinal function of the OFC (Ostlund and Balleine, 2008; Alcaraz et al., 2015). At a cellular level, however, it is not known whether the inputs from MD and the submedius thalamic nucleus actually converge on single cortical neurons. Convergence onto a single cortical cell could account for the synergistic amplification of signals, which may be particularly important for accentuating behaviorally relevant environmental features. The more general process of neuronal convergence would encourage the integration of different information streams, enabling the development of more detailed multisensory mental representations (Man et al., 2013).

Disentangling the functional contribution of diverging and converging thalamic inputs will be an important objective for future research, especially considering that these features of thalamocortical organization appear to be largely conserved between species (Padberg et al., 2009).

#### Subcortical integration

While interactions between the thalamus and cortex are critical for cognition, there is increasing evidence that thalamic nuclei may also have a role in integrating subcortical information. The ATn receives inputs from the hippocampal formation directly, mainly via the fornix (Dillingham et al., 2015b), as well as indirectly, via the mammillary bodies. However, this does not result in redundancy or a replication of information because the inputs are from distinct hippocampal populations (Amin et al., 2010; Christiansen et al., 2016), suggesting that the ATn may have a role in combining these separate hippocampal inputs. Projections from the mammillary bodies predominantly act as drivers to the ATn, whereas the cortical/hippocampal inputs have a modulatory role, specifically in the case of the anterodorsal nucleus (Somogyi et al., 1978; Petrof and Sherman, 2009). Consistent with this distinction between inputs, direct hippocampal inputs to the ATn elicit long-term depression, whereas the projections from the mammillary bodies elicit long-term potentiation (Tsanov et al., 2011a).

In addition to being a convergence point for separate hippocampal inputs, the ATn receives direct and indirect inputs from the midbrain tegmentum. The direct cholinergic input arises from the laterodorsal tegmental nucleus, whereas the indirect inputs come from the dorsal and ventral tegmental nuclei of Gudden. These midbrain inputs appear crucial for learning and memory (Mitchell et al., 2002; Taube, 2007; Vann, 2009; Clark et al., 2013; Vann, 2013), and the indirect inputs are critical for the head-direction and theta signals found in the ATn (Satoh and Fibiger, 1986; Bassant and Poindessous-Jazat, 2001; Kocsis et al., 2001; Bassett et al., 2007; Zakowski et al., 2017).

Other thalamic nuclei also exhibit a close partnership with subcortical areas, especially the striatum. The dorsal striatum is the recipient of a strong glutamatergic innervation originating from both the centro-median/parafascicular complex and the intralaminar group (Galvan and Smith, 2011). These thalamic groups are crucial for the role of the basal ganglia (BG) role in behavioral flexibility; they provide behaviorally relevant information and have been linked to goal-directed action selection in both rodents and humans (Brown et al., 2010; Kato et al., 2011; Liebermann et al., 2013; Schepers et al., 2017; Kato et al., 2018). For instance, removal of the parafascicular nucleus (Pf) in rats disrupted goal-directed behaviors by preventing rats from updating action-outcome associations (Bradfield et al., 2013b). Importantly, this manipulation was also found to reduce intrinsic activity of striatal cholinergic interneurons specifically, suggesting the existence of a direct link between behavioral flexibility and Pf inputs to these striatal cholinergic interneurons (Bradfield et al., 2013b; Yamanaka et al., 2018). Using cross-unilateral lesions of the Pf and striatum, Bradfield and Balleine (2017) showed that the impairment in updating action-outcome associations was due to an inability to use internal state to access the appropriate associations; in contrast, the use of external context to control accurate goal-directed action selection remained intact (Bradfield and Balleine, 2017). Lack of flexibility after inhibition of this thalamostriatal pathway is also evident in other paradigms, such as the five-choice serial reaction time task (Saund et al., 2017). These findings further highlight the importance of the thalamus in monitoring and updating mental representations in response to changing circumstances.

This latter consideration certainly calls for a critical assessment of how thalamocortical loops and the BG integrate. The thalamostriatal projection described above has only been minimally featured in classic models of BG functioning (Bostan and Strick, 2018). Instead, the emphasis has been on thalamic nuclei as a motor output, even though this does not address the contribution of thalamus-to-BG pathways (Goldberg et al., 2013). Such a model is supported by the fact that the motor thalamus, that is, the ventral anterior/ventral lateral thalamic areas, is one of the main recipients of BG output. But even this admittedly motor thalamic region has been shown to play a crucial role in performance monitoring (Seifert et al., 2011; Ullsperger et al., 2014).

The other main thalamic recipient of BG projections is the MD. This BG-MD pathway has also shown to be necessary for higher-order cognition (Leung and Balleine, 2015). As a result, a more integrative picture of the thalamus is now emerging, especially when considering the existence of multiple and interacting cortico-BG-thalamo-cortical loops (Haber and Calzavara, 2009; Bell and Shine, 2016). Interestingly, both corticostriatal and corticothalamic pathways were recently demonstrated to support behavioral flexibility, whereas corticocortical pyramidal prefrontal neurons were not (Nakayama et al., 2018), further demonstrating the key contribution of subcortical areas for high-order cognition. Together, these data suggest a critical role for the thalamus in integrating subcortical as well as cortical inputs to support cognitive functions.

#### The thalamus: a bridge between the medial temporal lobe and frontal cortex

To account for the myriad cognitive symptoms and memory impairments elicited by thalamic damage, an interesting early proposal was that the thalamus acts as a link between the medial temporal lobe and the frontal lobe (Warrington and Weiskrantz, 1982). Accordingly, disconnecting the hippocampal inputs to the ATn impairs performance on spatial memory tasks (Warburton et al., 2000). Furthermore, ATn damage in rodents and medial diencephalic damage in patients reduce activity in the frontal cortex (Kapur, 1994; Jenkins et al., 2002; Reed et al., 2003; Caulo et al., 2005; Vann and Albasser, 2009; Ozyurt et al., 2014). However, ATn lesion effects are unlikely to be due to the disruption of a one-way flow of information from the hippocampal formation to the frontal cortex as originally suggested. Indeed, the importance of the return projections from the ATn to the hippocampus has been recently highlighted (Vann and Nelson, 2015). For example, memory elicited event-related potentials in the ATn have been found to precede those in the hippocampus, suggesting a flow of information from ATn to the hippocampus, rather than the reverse (Stillova et al., 2015). The activity changes found in the frontal cortex following thalamic damage may therefore reflect a role for ATn in coordinating and synchronizing activity across hippocampal and cortical regions (Sweeney-Reed et al., 2014).

The MD could also be seen as link between the medial temporal lobe, as a recipient of projections originating from the BLA (technically a medial temporal lobe structure), and the OFC (Timbie and Barbas, 2015; Wolff et al., 2015a). It is therefore interesting to note that lesions to either MD or the BLA produce qualitatively different impairments when the contingency between predictive cues and their outcome is modified (Ostlund and Balleine, 2008). In that study, rats were trained to respond to two stimuli that reliably predicted a reward. Subsequently, one of the stimuli became noncontingently paired with reward, whereas the other remained a reliable predictor. After this manipulation, rats with BLA lesions did not adapt their behavior and continued to exhibit a positive conditioned response to both contingent and noncontingent stimuli. In contrast, rats with MD lesions exhibited a nonspecific effect with reduced responding to both the noncontingent and contingent stimuli (i.e., they ceased to respond to cues that still reliably predicted their outcome) (Ostlund and Balleine, 2008). This suggests that adaptive cognition requires the functional contribution of multiple complementary pathways. Further work is thus warranted to examine the functional contribution of the connections between these areas and their cortical target, the OFC.

The reuniens nucleus (Re) is another thalamic nucleus that has recently received much attention due to its role in connecting prefrontal and temporal lobe areas: it projects to multiple frontal areas and is the main source of thalamic afferents to the hippocampus. Because all of these projections are reciprocal, the Re is thought to be a major hub, orchestrating functional exchanges between frontal areas and the hippocampus, especially in the absence of direct inputs from the PFC to the hippocampus (McKenna and Vertes, 2004; Vertes et al., 2006; Hoover and Vertes, 2012; Varela et al., 2014). Because of these unique properties, an unusually large number of studies have been conducted recently to assess the possible functions of these Re projections. There are a number of excellent review papers available on the subject (Cassel et al., 2013; Cassel and Pereira de Vasconcelos, 2015; Pereira de Vasconcelos and Cassel, 2015; Vertes, 2015). Briefly, Re dysfunction affects contextual fear memory (Xu and Sudhof, 2013; Vetere et al., 2017), impairs spatial memory consolidation (Loureiro et al., 2012), either increases or decreases behavioral flexibility (Cholvin et al., 2013; Linley et al., 2016; Prasad et al., 2017; Viena et al., 2018), impedes goal-directed spatial navigation (Ito et al., 2015), or delayed nonmatching-to-place (Hallock et al., 2013, 2016; Viena et al., 2018), and even affects visual threat responses (Salay et al., 2018). Given these diverse findings, continued research is needed before it is possible to generate global functional models of this region. Nevertheless, an interesting proposal, and a recurring theme across thalamic nuclei is that the Re may be particularly important for synchronizing PFC and hippocampal activity, facilitating functional exchanges between these areas (Eichenbaum, 2017). The wide array of functions that appear to be Re-dependent may therefore reflect the diverse nature of the tasks that require functional cooperation between PFC and hippocampal areas.

### Future directions

**Figure 3.**
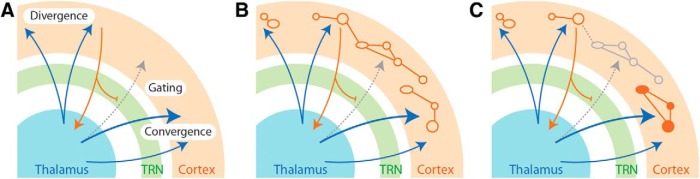
***A***, Divergence, convergence, and gating are essential functional principles of thalamocortical circuits. Divergent and convergent thalamocortical pathways may promote parallel functioning and integrative processing at the cortical level, respectively. In addition, returning corticothalamic pathways are able to gate relevant thalamocortical inputs through lateral inhibition at the level of the TRN. This architecture may contribute to the maintenance (***B***) or updating (***C***) of cortical representations. In the latter case, some mental constructs may no longer be relevant (outlined in gray dashed line), whereas others become more prominent (blue solid arrows).

At this point, it should be evident that cognitive functions are supported by distributed neural circuits among which thalamic nuclei play highly integrative roles. In this *Viewpoints* article, we examined recent evidence implicating thalamic nuclei in high-order cognitive functions and have attempted to highlight how many of these new findings support aspects of early cognitive views of thalamic functions (e.g., Warrington and Weiskrantz, 1982; Sherman and Guillery, 1996; Aggleton and Brown, 1999). We therefore propose that a central role for the cognitive thalamus is to shape mental representations, either by maintaining relevant mental constructs online or by updating those no longer relevant for ongoing challenges (Fig. 3). This proposition builds on many diverse experimental findings and focuses on general thalamic functions rather than specific contributions of individual nuclei.

The thalamus has been increasingly implicated in neurological disorders that present with cognitive dysfunction, including schizophrenia (Pinault, 2011; Uhlhaas et al., 2013; Anticevic et al., 2014), drug addiction (Balleine et al., 2015), Korsakoff syndrome (Victor et al., 1971; Harding et al., 2000), Alzheimer's disease (Braak and Braak, 1991), and Down syndrome (Karlsen et al., 2014; Perry et al., 2018). Therefore, new models of thalamic function may better explain the pattern of deficits associated with these conditions.

While we share the belief that thalamus research has entered a new era and that virtually any brain region has a thalamic story to tell (Acsády, 2017), we also anticipate the danger of being overwhelmed by data that may be difficult to replicate or to extend. Relying on standardized behavioral assays may be key, as is in-depth analysis of the literature. Technical developments progress at a much higher rate than conceptual advances. The latter can only be supported by a better analysis of how new data integrate with the preexisting knowledge. In doing so, conceptual views may be enriched, revised, or even drastically changed if novel technical approaches are used to test some of their predictions.

As discussed at several points throughout this article, there are areas of thalamic research where our current knowledge is woefully lacking. Filling these missing gaps could prove invaluable in testing and advancing current models of thalamic function. First, the functional relevance of divergent and convergent thalamocortical or corticothalamic pathways needs to be addressed more systematically. Until recently, this has been technically impossible due to methodological limitations. But by using a combination of chemogenetic and/or optogenetic approaches, it is now possible to test the relative contributions of these separate pathways. There is no single, definitive experiment to address this issue; instead, we believe the combined effort across multiple laboratories will be essential to advancements in this area. Second, although a large part of this *Viewpoints* article has emphasized functional interactions between the cortex and the thalamus, we also highlighted the importance of the thalamus for subcortical integration. There is a clear need for additional data to better appreciate how the thalamus interacts with the BG and other subcortical areas. Furthermore, the advances in multisite *in vivo* recordings will help address the contribution of the thalamus for coordinating and synchronizing brainwide activity. Finally, work on the thalamus has been performed either by researchers primarily interested in sensory-motor nuclei or by those interested in cognition. Surprisingly, these two worlds have been quite separate and relatively unaware of the progress made in the other field. We believe in the potential of uniting efforts in promoting a cognitive view of the thalamus, from sensory salience to adaptive cognition.
